# Editorial: Molecular Engineering of Sensory Mechanisms in Bacteria for Biosensing Technologies and Novel Tools for Microbial Engineering

**DOI:** 10.3389/fbioe.2022.894553

**Published:** 2022-04-25

**Authors:** Rodolfo García-Contreras, Toshinari Maeda, Bernardo Franco

**Affiliations:** ^1^ Laboratorio de Bacteriología, Departamento de Microbiología y Parasitología, Facultad de Medicina, Universidad Nacional Autónoma de México, Mexico City, Mexico; ^2^ Department of Biological Functions Engineering, Gradute School of Life Sciences and System Engineering, Kyushu Institute of Technology, Kitakyushu, Japan; ^3^ Departamento de Biología, División de Ciencias Naturales y Exactas, University of Guanajuato, Guanajuato, Mexico

**Keywords:** synthetic biology, regulatory mechanisms in bacteria, biosensing technology, genetic engineering, cell-to-cell communication, artificial sensory pathways in microorganisms

Synthetic biology has become a novel and exciting field in biology, opening many possibilities for engineering cells for out-of-the-box solutions in many fields.

After learning how to ‘read’ DNA, we can ‘write’ it so that molecules and organisms can perform new tasks. The tools and methods developed for creating individual molecules or whole genomes have paved the avenue for constructing complete genomes and designing organisms capable of producing specific molecules, identifying essential genes, redesigning metabolic pathways, and many others (Ostrov et al., 2019). This has been possible by the profound knowledge of genomes, transcriptomes, and proteomes of different organisms, leading to more comprehensive databases that set the foundation for organisms and genetic circuit designs.

Also, this technology is getting closer to assessing a relevant question: what is the minimal set of genes required for life to thrive? The available minimal genomes, such as JCV-syn3.0 (Hutchison et al., 2016) uncover a set of genes that at first sight seem to be dispensable since all of them were of unknown function. Also, the future for genome design and synthesis may rely on computer-assisted simulations that are getting closer to modeling the behavior of complete cells (Rees-Garbutt et al., 2020), allowing more comprehensive studies for assessing gene function and organism evolution.

**FIGURE 1:**
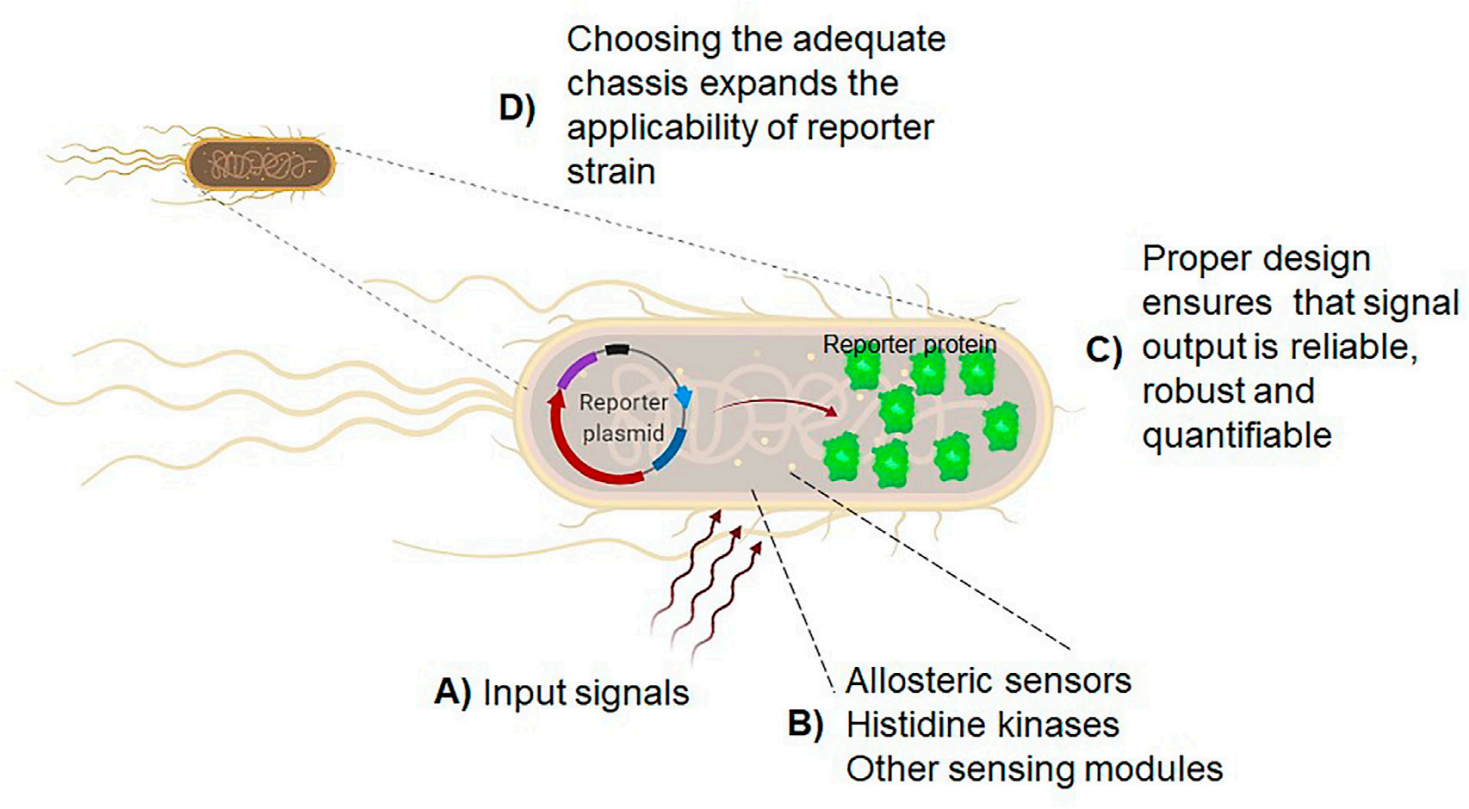
Biosensors using whole cells are versatile and, depending on the chassis, be resilient to severe conditions. The framework for biosensor development includes clear research goals, knowing the literature for assessing the limiting steps in a metabolic or regulatory pathway, identifying the biological parts to be repurposed and their characterization, proof-of-concept checkpoint at laboratory scale, and finally, the development stage (Moratti et al.). In **(A)**, the selection of input signals is the first step to determine the goal of the whole-cell reporter strain. In **(B)**, as described by Famerling et al. the identification of allosteric sensors, histidine kinases or other sensing modules is relevant for designing reporter strains. Novel sensing modules can be repurposed with the current knowledge in many culturable or unculturable organisms. In **(C)**, as described by Wang and Childers, Moratti et al., and Zhang et al., optimizing the design that ensures signal perception and output is specific, sensitive, reliable, and robust. Finally, as reported by Tietze et al., in **(D)**, the selection of the cell chassis and the optimization of the regulatory elements ensures that the biosensor will fulfill the optimal characteristics for biosensing technologies in the future.

On a smaller scale, the use of synthetic biology methods has made it possible to harness the power of the inducible responses of bacterial, yeast, and other organisms, to develop biosensors (Figure 1). This technology has been directed to different applications, from single molecules to whole cells, from the engineering of biosynthetic pathways (Rogers et al., 2016) to environmental monitoring (Elad and Belkin, 2017).

This Research Topic contains six contributions in the field of biosensing technologies that begins with a relevant topic: the sensory systems in a model bacterium (*Escherichia coli*), knowing the catalog of sensing and transcriptional regulatory network allows to design desired application in this model organism, and can also be expanded to other organisms. Famerling et al. contributed to this topic with a systematic review covering the regulatory network in *E. coli*. The priceless contribution to our understanding of regulatory networks contained in RegulonDB, the largest and most comprehensive data repository accessible to all. The authors provided a framework for defining regulatory sensing mechanisms such as GENSORS or elementary Genetic-Sensory-Response units to define the collection of genetic response elements that ultimately lead to the design of genetic circuits based on the feedback different GENSORS provide. In this review also, novel regulatory networks are reported.

In this topic, Wang and Childers provided a mini-review article that sets biosensing technology in the spotlight of current applications of this technology: the potential of biosensors to investigate the gut-brain axis Wang and Childers. Many human behavioral features are related to the gut microbiome due to the intimate communication between microbial organisms residing in the gut with the brain and vice versa. In this minireview, the authors pinpoint several biomarkers, such as tetrathionate, tryptophan, indole as metabolic markers, for biosensor-based detection. Also, the design of methods for determining inflammation may address the role of dysbiosis or gut-resident microbes that may help uncover potential markers for depression. The authors hypothesize that gut microbes may hold novel sensory systems that may be exploited for biosensing technology.

On another scale, Moratti et al. contributed another contribution of biosensors: environmental pollution detection. Monooxygenases are key enzymes for the degradation of alkanes and alkenes, and their regulation is a key for repurposing these systems to achieve biosensors for the detection of these important pollutants. The authors provide a comprehensive framework for developing biosensors that are aimed to detect hydrocarbons, including the current knowledge and the future perspectives for generating different chassis for sensing hydrocarbons. Importantly, the present technology should be expanded to other hydrocarbons and explore the unexploited riches found in genome sequences from yet-to-be-cultured bacteria.

Following the same line of the applicability of biosensors, Zhang et al. provided an excellent example of a reporter strain developed in *Pseudomonas putida* KT2440 with higher sensitivity and specificity to cadmium. This was achieved by generating a feedback circuitry that enhanced the sensitivity of this biosensor by 33% and increased sensitivity by 400-fold, therefore being capable of detecting a lower limit that the World Health Organization established for drinking water. This report is an excellent example where good design, and the correct cellular chassis may render specific and tunable biosensor strains. This biosensor may be applied regularly to monitor water pollution with the specificity and sensitivity shown.

Kim et al. provided another framework for developing biological parts for biosensing technology: the selection of transcription factor-promoter pairs with improved dynamic range for the detection of butanol. This paper highlights the future for biosensors: coupling high throughput technologies for selecting or improving genetic regulatory sequences can ultimately improve the detection range of the biosensor. This report used sort-seq to enhance the detection limit for the P_BMO_ promoter from *Thauera butanivorans*, implemented in *E. coli*. This shows that using novel approaches such as FACS and Next Generation Sequencing, existing promoter sequences can be further optimized for homologous or heterologous systems. This innovative approach may help develop novel regulatory strategies for other chassis.

The recipient cell machinery may impede the use of heterologous biological parts. In the experimental approach, Tietze et al. novel promoter sequences were identified that could be used in the fast-growing bacterium *Vibrio natrigens*. Also, the dependence on the Shine-Dalgarno sequence seems stringent in this organism and should be considered in designing synthetic regulatory 5’ sequences. Some promoters identified in this study were species-specific, suggesting that in this case, further analysis is needed to establish the potential differences in regulatory mechanisms, something to consider when optimizing regulatory sequences in different organisms. Here is another example of a framework to further optimize and create novel regulatory elements for biosensing applications using different cellular chassis.

The high quality of papers presented in this topic positions biosensing technologies in the spotlight for further developing biosensors with novel applications. In the near future, these technologies may become a gold standard for detecting different molecules and solving many health, industrial, and ecological problems issues.
